# Optimizing Telehealth Experience Design Through Usability Testing in Hispanic American and African American Patient Populations: Observational Study

**DOI:** 10.2196/16004

**Published:** 2020-08-04

**Authors:** D'Arcy King, Sundas Khan, Jennifer Polo, Jeffrey Solomon, Renee Pekmezaris, Negin Hajizadeh

**Affiliations:** 1 Donald and Barbara Zucker School of Medicine at Hofstra/Northwell Manhasset, NY United States

**Keywords:** chronic obstructive pulmonary disease, usability testing, telehealth, telerehabilitation, vulnerable populations

## Abstract

**Background:**

Telehealth-delivered pulmonary rehabilitation (telePR) has been shown to be as effective as standard pulmonary rehabilitation (PR) at improving the quality of life in patients living with chronic obstructive pulmonary disease (COPD). However, it is not known how effective telePR may prove to be among low-income, urban Hispanic American and African American patient populations. To address this question, a collaborative team at Northwell Health developed a telePR intervention and assessed its efficacy among low-income Hispanic American and African American patient populations. The telePR intervention system components included an ergonomic recumbent bike, a tablet with a built-in camera, and wireless monitoring devices.

**Objective:**

The objective of the study was to assess patient adoption and diminish barriers to use by initiating a user-centered design approach, which included usability testing to refine the telePR intervention prior to enrolling patients with COPD into a larger telePR study.

**Methods:**

Usability testing was conducted in two phases to identify opportunities to streamline and improve the patient experience. The first phase included a prefield usability testing phase to evaluate technical, patient safety, and environmental factors comprising the system architecture. This was followed by an ergonomic evaluation of user interactions with the bicycle, telehealth tablets, and connected wearable devices to ensure optimal placement and practical support for all components of the intervention. The second phase of research included feasibility testing to observe and further optimize the system based on iterative rounds of telePR sessions.

**Results:**

During usability and feasibility research, we identified and addressed multiple opportunities for system improvements. These included physical and environmental changes, modifications to accommodate individual patient factors, safety improvements, and technology upgrades. Each enrolled patient was subsequently identified and classified into one of the following 3 categories: (1) independent, (2) intermediate, or (3) dependent. This categorization was used to predict the level of training and support needed for successful participation in the telePR sessions. Feasibility results revealed that patients in the dependent category were unable to perform the rehab sessions without in-person support due to low technical acumen and difficulty with certain features of the system, even after modifications had been made. Intermediate and independent users, however, did exhibit increased independent utilization of telePR due to iterative improvements.

**Conclusions:**

Usability testing helped reduce barriers to use for two subsets of our population, the intermediate and independent users. In addition, it identified a third subset, dependent users, for whom the telePR solution was deemed unsuitable without in-person support. The study established the need for the development of standard operating procedures, and guides were created for both patients and remote respiratory therapists to facilitate the appropriate use of the telePR system intervention. Observational research also led to the development of standard protocols for the first and all subsequent telePR sessions. The primary goals in developing standardization protocols were to establish trust, ensure a positive experience, and encourage future patient engagement with telePR sessions.

## Introduction

Chronic obstructive pulmonary disease (COPD) is a disease that occurs when airflow to the lungs is obstructed, and it is classified as chronic inflammatory lung disease caused by exposure to irritating gases or particulate matter (most commonly cigarette smoke). If the disease is not treated, symptoms often get worse due to excessive inhalation of irritating gases, and by the time these symptoms appear, significant lung damage has already occurred [1]. Acute COPD exacerbations lead to further loss of lung function, are associated with decreased quality of life (QoL) and increased morbidity and mortality, and generate a significant cost to the health care system [2-4]. Current data suggests that COPD mortality is increasing, and COPD is presently the third leading cause of death in the United States, claiming 134,676 lives in 2010 [5]. In addition, an estimated 715,000 hospital discharges related to COPD were reported in 2010, which is a discharge rate of 23.2 individuals per 100,000 population; of these discharges, 65% were 65 years or older [6].

Health and healthcare disparities have been observed among ethnic minority populations, with African American and Latin American populations showing more rapidly rising death rates than the non-Latin white American population. Both African American and Hispanic American patients bear a high burden of illness and death due to COPD and are twice as likely to visit the emergency room for COPD-associated conditions as compared to non-Hispanic American white patients [7,8]. Higher rates of smoking, reduced healthcare access, and lower socioeconomic status all contribute to this high disease burden in both African American and Hispanic American patients [9]. Patients admitted for COPD exacerbation have a 23% risk of 30-day readmission and a 50% risk of 12-month readmission, and both African American and Hispanic American race/ethnicity are associated with an almost twofold increase in hospitalization risk [10,11].

**Figure 1:**
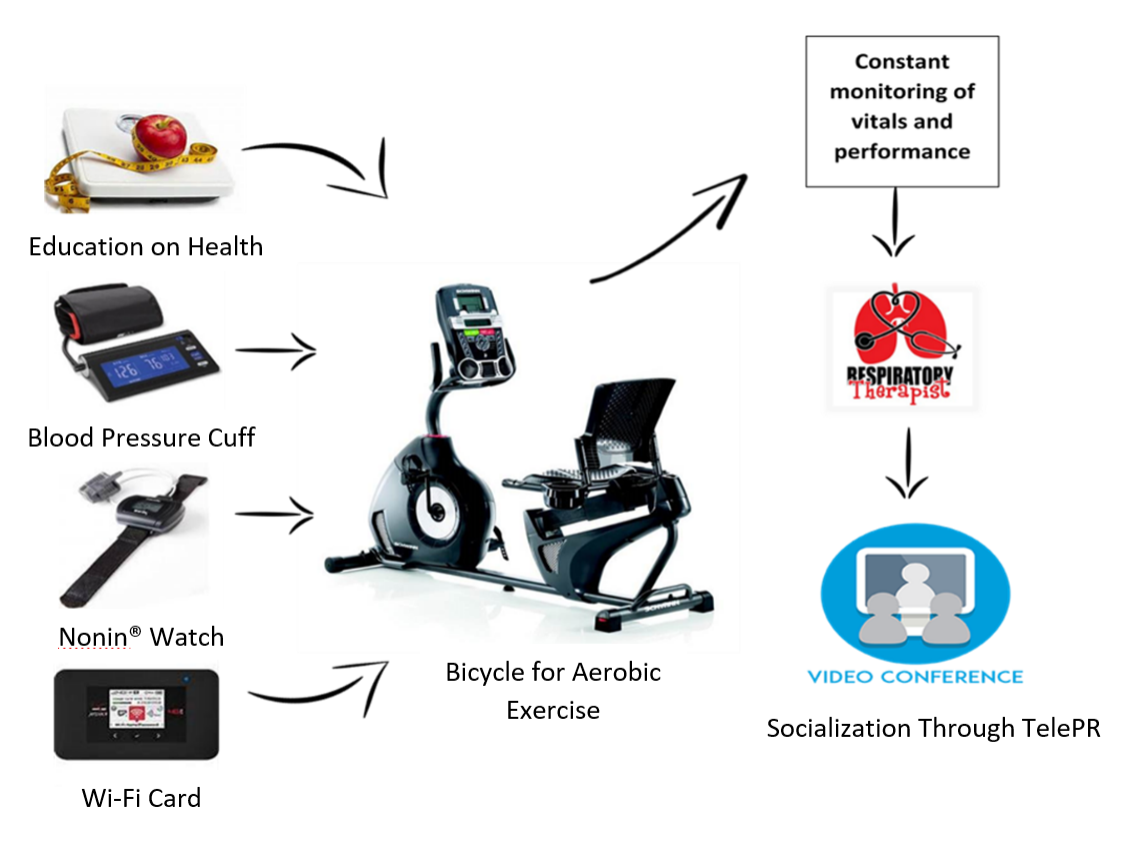
Telehealth-delivered pulmonary rehabilitation (telePR) model of care.

Early pulmonary rehabilitation following hospital admission has been shown to improve QoL and to decrease readmissions [12]. Telehealth-delivered pulmonary rehabilitation (telePR) has been shown to be as effective as standard pulmonary rehabilitation (PR) at improving QoL [12,13]. However, it is not known how effective telePR will be among low-income, urban Hispanic American and African American populations [14]. To assess outcomes in these populations, we developed a telePR intervention that included an ergonomic recumbent bike with graded exercise levels, a tablet with a built-in camera, and wireless monitoring devices designed for use in patients’ homes and local community centers, as illustrated in Figure 1. The goal of the initiative was to improve the management of patients with COPD in disparity populations by providing point-of-care services accessible through telePR in the community and patients’ homes.

The primary goal of the study was to compare the effectiveness of a referral to telePR versus standard PR for patients hospitalized for COPD exacerbation. Although there are data on standard PR improving outcomes in Hispanic American and African American patients, patients from these populations are not included in studies exploring the efficacy of telePR, despite evidence that underserved Hispanic American and African American patients have positive perceptions of telehealth interventions in general [15,16]. Barriers to access that disproportionately affect disparity patients include lack of referral to PR due to perceived ineffectiveness; lack of insurance coverage or high copayments; and difficulty accessing PR due to transportation costs, distance, and lack of caregiver support. This study aimed to overcome many of these major barriers by providing PR outside of the standard PR setting, via telehealth settings. Participants had the option of choosing to receive telePR either within the patient’s home or in a community center.

We sought to identify opportunities to streamline and improve the patient experience in different settings (either in a community center or in a patient’s home) prior to recruiting and enrolling patients with COPD into a larger study. In the larger study, patients participated in telePR for 1-hour sessions that occurred 2 times a week for 8 weeks, and who were subsequently followed for 1 year. The objective was to encourage patient adoption and to remove barriers to use by adopting a user-centered design approach to refine the telePR intervention system during prefield testing, as well as to conduct usability testing for select patients who were having difficulty with the telePR technology.

## Methods

### Study Design

Usability testing was conducted in two phases to identify opportunities to streamline and improve the patient experience by evaluating technical, safety, and environmental factors. Phase 1 included a prefield usability testing assessment that consisted of (1) design ergonomics for optimal placement of the bicycle, telehealth tablets, and connected wearable devices; (2) user-centered design optimizations based on real-world observations of users; and (3) development and documentation of standards of operation (SOPs) for all subsequent sessions. Phase 2 included in situ observation of patients during telePR sessions and assessment of their level of engagement with the remote respiratory therapist. Prior to the commencement of research activities, approval was obtained from Northwell Health’s Institutional Review Board. Patients who agreed to enroll in the usability and feasibility testing were invited to complete the Northwell Health audiovisual recording authorization form prior to fielding.

### Recruitment Methods

Participants recruited for the usability testing included patients hospitalized for COPD exacerbation at Northwell Health and Wyckoff Heights Medical Center. The patients recruited from Northwell Health were drawn from one of 7 Northwell hospital locations: Long Island Jewish Medical Center, North Shore University Hospital, Long Island Jewish Forest Hills, Southside Hospital, Glen Cove Hospital, Huntington Hospital, or Long Island Jewish Valley Stream. The source of referrals was inpatient admissions to the targeted hospitals. Patients were also recruited from their homes or outpatient doctors’ offices immediately after discharge (up to 2-3 weeks post-hospital discharge). Feasibility was assessed by querying databases at all 8 hospitals for COPD and stratifying by race/ethnicity. Race/ethnicity was classified according to participants’ self-identification. Based on the responses from a needs assessment, all of the Hispanic American and African American patients with severe COPD who were asked whether they would participate in the telePR system expressed interest.

Eligible patients were approached for consent to participate in the usability session. A session included the following activities: two brief usability questionnaires to collect information about attitudes and experiences with the rehab session, a postinterview to inquire about experiences during the usability session, and audio recordings of the session. Informed consent included a detailed description, in English and Spanish, of the risks and benefits of the study with user-friendly images of the equipment.

For Phase 2 (the feasibility phase), 4 participants were enrolled. This is a typical sample size for usability studies, as prior studies have elicited a sufficient response of usability issues [17-19]. The demographics of the participants are outlined in Table 1.

**Table 1:** Participant demographics (N=4).

Participants	Hospital	Age	Race/Ethnicity	Gender
1	Long Island Jewish Forest Hills^a^	81	Hispanic American	Male
2	Long Island Jewish Medical Center^b^	87	African American	Male
3	Long Island Jewish Forest Hills^a^	63	African American	Male
4	Long Island Jewish Medical Center^b^	71	Hispanic American	Female

^a^Community hospital.

^b^Large academic center.

### Statistical Analysis

To ensure the acceptability and usability of the telePR, we used a mixed methods approach to look at indicators of usability and acceptability in two stages. First, we analyzed usability testing sessions using qualitative analytic methods. All usability testing sessions were audio-recorded and professionally transcribed. Structural coding was used to mark responses to topical questions in the usability questionnaires. The data were categorized to develop a codebook and independently coded by 3 coders. The main themes that emerged indicated necessary adaptation to increase the usability and acceptability of the telePR system. Second, we measured whether participants were able to complete the usability sessions, using quantitative methods (eg, system usability scale) and qualitative measures (eg, identification of any technical or logistical barriers encountered).

The usability questionnaire assessed the following elements:

1. Comfort level with physical elements such as the seat, screens, blood pressure monitor, and pulse oximetry
2. Experience and interaction with the respiratory therapist throughout the session
3. Ability to see the rehabilitation video during the session
4. Effective visualization of co-participants during the session
5. Overall experience with the bike and all of its components

### Phase 1: Prefield Testing Assessment

**Figure 2:**
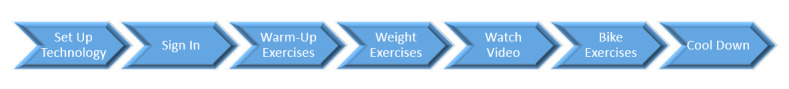
Telehealth-delivered pulmonary rehabilitation (telePR) session outline.

Phase 1 included a prefield testing assessment of the telePR system consisting of technical, safety, and environmental factors. This was followed by an ergonomic evaluation of user-interaction with the bicycle, telehealth tablets, and connected wearable devices to ensure optimal placement and practical support for all components of the intervention. A key output of the prefield testing assessment was the development of a user manual based on the user-centered design and ergonomics assessment. Protocols were developed to ensure personalized requirements were met prior to the start of each telePR session (see Figure 2). These included adjusting the seat height of the bicycle and tablet screen; changing of gears; setting the best audio levels; and placement of weights and bands, rescue inhaler, water, food for patients with diabetes, or other items needed in case of an emergency (such as a mobile phone). At the start of each telePR session, the remote respiratory therapist was required to record baseline measurements including blood pressure, pulse rate, pulse oximetry, and glucose (in patients with diabetes), which were assessed through connected wearable devices. Part of the ergonomics assessment was to ensure these wearable devices were placed in areas easily accessible to patients throughout the session. Usability testing was conducted face-to-face with the patient and remotely with the respiratory therapist to best understand the patient experience.

The remote respiratory therapist was a key player in the telePR session and set the tone of the experience. As part of the output from the initial prefield testing assessment, an SOP manual was developed for the therapist. The guide included instructions for the therapist to review with the patient prior to each session. These included an equipment checklist, instructions for how and when to check the patient’s vital signs, prompts for transitioning from one activity to another, recommendations for a personalized script used at the opening and close of each session, and guidance for wrapping up each session with the patient. A quick-access checklist was also developed for the respiratory therapist as a reminder to adjust monitor screens to patient eye level prior to the session; to constantly observe the patient throughout the session; to adjust volumes for the patient during specific parts of the session (eg, for the educational video); and to prompt time considerations for specific tasks. Safety protocols were also developed to assess for issues and to troubleshoot situations in which loss of video or wireless connection occurred, patients had trouble using the bike or other equipment, specific tablet-related issues (eg, pop-up on the screen) occurred, or any emergencies arose (eg, the patient falls during the session). Usability testing was conducted through mock-session simulations with the respiratory therapist before the implementation of the feasibility testing.

### Phase 2: Feasibility Testing

Feasibility testing was performed to assess ease of use and usefulness of each telePR component, including the bicycle, tablet, and wearables during a 90-minute telePR session. Real-world usability testing sessions were conducted with patients to uncover obstacles in workflow that were unable to be identified during simulated usability testing sessions. With the support of a research study member, 4 encounters were observed between a single patient and a remote respiratory therapist. Of these 4 observed encounters, 1 encounter took place at a community center and the other 3 encounters took place within patient homes. We began each session by scanning the different types of locations to assess possible issues with the environment and physical space. Audio recordings of the participant and respiratory therapist were made during each session. Following the telePR session, patients were asked to complete two surveys: (1) a set of usability questions for a thorough review of their experience and opinions regarding the telePR session, and (2) the widely validated 10-question System Usability Scale (SUS) [20].

## Results

### Phase 1: Prefield Testing Assessment

**Figure 3:**
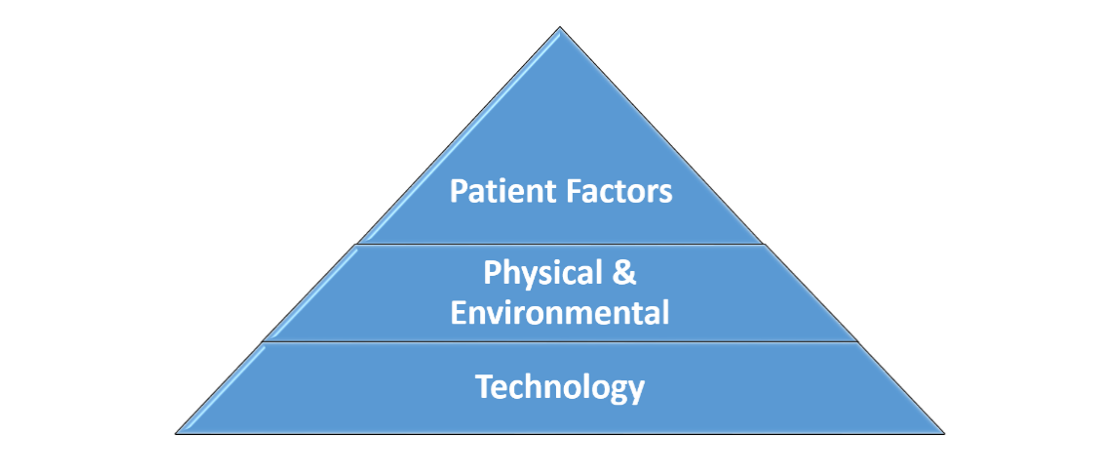
Fundamental assessment prioritization scale.

A fundamental assessment prioritization scale (see Figure 3) was configured based on the initial observations of the telePR sessions to account for technical issues, physical and environmental obstacles, and potentially problematic patient factors. Technical issues were assessed and quickly modified at the start of each session, as bandwidth and other issues caused considerable delays and confusion on the part of the patient, leading to demotivation and frustration. If a strong internet connection could not be established, the session could not be completed. With regard to the ergonomics of the tablet, many patients experienced difficulty with interface issues, such as trouble with double-clicking icons that were deemed too small (especially for those with arthritis and those with visual impairment). Tablet placement needed to be assessed based on patient mobility, vision needs, and auditory needs—this was a critical step that required a research study team member to make requisite device adjustments between the different patient visits to the community center.

When considering environmental and physical issues, we found through usability testing that it was vital for the remote respiratory therapist to review a checklist with the patient to ensure the following items were present prior to the session: water, oxygen (if necessary), weights, and a phone in case of an emergency, in order to facilitate participant independence during subsequent sessions. Prior to the encounter, the SOP manual also called for the therapist to advise patients to adjust the angle of the tablet and position the seat for the most advantageous level of comfort. During this phase of usability testing, the need to modify the tablet mounting was identified as a priority optimization to prevent patient neck strain when viewing the tablet, riding the bike, and completing other exercises during the session. Over the course of the study, additional opportunities for system improvements were found and implemented, which included physical and environmental changes, individual patient factor accommodations, safety improvements, and technology modifications (Table 2).

**Table 2:** Summary of system improvements.

Issue			Modification
**Physical/environmental**			
	Patients that have low motor and upper body strength have trouble adjusting the bike seat to a level of comfort and the tablet to eye level.	Prior to the encounter, the respiratory therapist will need to advise patients to adjust the angle of the tablet and the seat position to the most advantageous comfort level. In certain patients, the study team member will need to be present to adjust the seat.	
	Tablet mounting arm is too short and may cause neck strain for certain patients.	Tablet mounting should be on a longer arm stand to prevent patient neck strain when viewing the tablet, riding the bike, and completing other exercises during the session.	
	The lack of a standardized approach to each telePR^a^ session leads to patient dependency.	The respiratory therapist should present a checklist to the patient prior to the encounter to check items (such as the seat, tablet, water, oxygen, weights, phone, etc) so that the patients may become independent during subsequent sessions.	
**Safety**			
	Emergencies that occur during the encounter must be addressed.	Develop a standard of operations for patient emergencies that may occur during the telehealth visit.	
	Wi-Fi may be lost during the telePR session.	Handouts should be available with directions for patients to answer the phone or to call the respiratory therapist if Wi-Fi is lost.	
	Patients that have knee problems may have trouble completing the telePR session.	Consider mobility criteria prior to a patient’s enrollment in the study (eg, patients who have had a recent knee surgery may encounter difficulties riding a bike).	
**Technology**			
	Bandwidth issues cause considerable interruptions throughout the session.	An initial technical assessment is needed at each site to ensure that Wi-Fi is sufficient to support telehealth technology. If issues continue to arise, then a research study member should be present at a particular site to address them.	
	Audio problems may occur during a telePR session.	In case of audio problems during teleconferencing, the patient’s cell phone number should be provided to the respiratory therapist to call if the teleconference goes down.	
	The Windows software has small close (X) icons that are not ideal for patients with large fingers, arthritis, or visual impairment to exit screens.	For users in the Dependent category, the technical demands required to configure and use the tablet hardware, software, and the associated devices are too tedious for a successful experience; these patients require a study team member to be present at the telePR session.	

^a^telePR: telehealth-delivered pulmonary rehabilitation.

### Phase 2: Feasibility Testing

After the implementation of the usability recommendations, two rounds of feasibility testing were conducted with 4 patients (2 in the community center setting and 2 in the patients’ homes). Upon completion of the telePR session, each patient completed one SUS inventory for the bike and one SUS inventory for the technology. Patients were asked to answer questions on a Likert scale from 1-5, with 1 coded for “Strongly disagree” and 5 coded for “Strongly agree.” Answers were converted, added, and multiplied by 2.5 to convert scores from a 0-40 scale to a 0-100 scale. Scores of 70 and above were considered acceptable while scores of 85 and above were considered excellent. Of the 4 patients, 3 completed the SUS. For the bike SUS, patient 1 scored 95% (38), patient 2 scored 50% (20), and patient 3 scored 25% (10). For the technology SUS, patient 1 scored 90% (36) and patient 2 scored 52.5% (21); patient 3 did not complete the technology SUS.

After completing two rounds of feasibility testing, 3 different categories of patients were classified as follows: independent, intermediate, and dependent (Table 3). This categorization was assessed and applied for each patient for all subsequent telePR sessions, used to predict the level of training and support needed for successful participation in all future sessions. Results revealed that those in the independent category could manage the telePR sessions by themselves. We determined that patients in the intermediate category required assistance during their initial sessions but were able to complete subsequent rehab sessions without support. Respondents in the dependent category were unable to perform the rehab sessions without in-person support due to low technical acumen and difficulty with certain features of the system, such as not being able to locate the icon to start the session, connect the tablet to the Wi-Fi, turn on the tablet, etc. The intermediate and independent users, however, did exhibit increased independent utilization due to iterative improvements to the system architecture and greater technical acumen.

**Table 3:** Telehealth-delivered pulmonary rehabilitation (telePR) participant categorization.

Category	Characterization
Independent	Most likely to be able to complete the telehealth rehab session without in-person support (other than initial set-up instructions).
Intermediate	Requires in-person support for the initial 2-3 telehealth rehab sessions but is able to independently complete the remaining sessions.
Dependent	Unable to perform the telehealth rehab sessions without in-person support.

## Discussion

The goal of usability testing is to identify potential barriers and develop recommendations for optimizing applications like the telePR program. The primary technical recommendation derived from our usability testing was to assess Wi-Fi bandwidth as part of the set-up protocol and installation process. Following this recommendation, we developed patient-friendly troubleshooting guidelines, including audio-only communication options to be used in cases where Wi-Fi issues occurred during the session.

Safety recommendations included the development of SOPs for patient emergencies that may occur during the telePR visit. Usability testing and user-centered design practices helped to identify a need for remote respiratory therapist guidelines and a readiness checklist for patients to review prior to commencing each telePR rehab session. This process also highlighted the necessity of developing protocols and operating standards to address technical, safety, and environmental issues. Protocols and SOPs included a diagram with seat, tablet, water, oxygen, weights, and phone placements; a sticker to remind patients to answer a phone call from the respiratory therapist in case of lost Wi-Fi; and a checklist for patients with mobility issues. Physical and environmental recommendations included the creation of markers (with the use of colored tape) at the initial visit that indicate personalized settings for the tablet angle, seat placement, and audio levels appropriate for the environment. The study team was also prompted to ensure the provision of power strips with each bike in case more than one electrical cord was needed (for the bike and tablet).

We further identified the need to assess for technical acumen at the first telePR session in order to categorize patients into Independent, Intermediate, and Dependent segments. This assessment allowed for appropriate study personnel to be deployed to the telePR visits and for specific, tailored instructions to be used. For users in the Dependent category, the technical demands required to configure and use the tablet hardware, software, and the associated devices made it impossible to conduct sessions successfully without the presence and aid of a research study team member. It was also important to interpret the results of the SUS to effectively place patients into the Telehealth-delivered pulmonary rehabilitation (telePR) participant categorization (Table 3), as patients in the Dependent category rated the bike and technology with scores of 50 and below. The primary goals in developing standardization protocols were to establish trust, ensure a positive experience, and encourage future patient engagement with telePR sessions. Our usability testing allowed us to achieve our primary goal and create a feasible protocol to support and guide current and future telePR session participants.

## Abbreviations

COPD: chronic obstructive pulmonary disease
PR: standard pulmonary rehabilitation
QoL: quality of life
SOPs: standards of operation
SUS: System Usability Scale
telePR: telehealth-delivered pulmonary rehabilitation
